# Retinal sensitivity assessed by microperimetry and its topographic correlation with macular structure by optical coherence tomography following macular hole surgery: a cross-sectional study

**DOI:** 10.1186/s40942-026-00904-4

**Published:** 2026-07-20

**Authors:** Rodrigo Schwartz Pegado, Gabriel Castilho Sandoval Barbosa, Luciana Virgínia Ferreira Costa-Cunha, Leandro Cabral Zacharias, Rony Carlos Preti, Mário Luiz Ribeiro Monteiro, Leonardo Provetti Cunha

**Affiliations:** 1 Instituto Brasileiro de Assistência e Pesquisa em Oftalmologia (IBAP Oftalmologia), Niterói, RJ Brazil; 2 Department of Ophthalmology, Hospital das Clínicas, Faculty of Medicine, University of São Paulo (FMUSP), São Paulo, SP Brazil; 3 Juiz de Fora Eye Hospital (Hospital de Olhos Juiz de Fora — HOJF), Juiz de Fora, MG Brazil; 4 Department of Ophthalmology, Faculty of Medicine, Federal University of Juiz de Fora(UFJF), Av. Eugênio do Nascimento, s/n — Dom Bosco, Juiz de Fora, MG 36038-330 Brazil

**Keywords:** Macular hole, Internal limiting membrane peeling, Microperimetry, Retinal sensitivity, Optical coherence tomography, Ganglion cell complex, Structure-function correlation

## Abstract

**Background:**

To evaluate retinal sensitivity (RS) assessed by microperimetry in the macular region of patients undergoing surgical repair of idiopathic macular hole (MH), and to investigate the topographic correlation between RS and both total macular thickness (TMT) and ganglion cell complex (GCC) thickness obtained by optical coherence tomography (OCT), establishing point-by-point anatomic correspondence between sensitivity measurements and their structurally matched retinal areas, six months after successful MH surgery with internal limiting membrane (ILM) peeling.

**Methods:**

Observational, analytical, cross-sectional study including 40 consecutive patients who underwent 25-gauge pars plana vitrectomy with ILM peeling for idiopathic MH at a single center (Juiz de Fora Eye Hospital, Brazil) between 2022 and 2024, with a minimum postoperative follow-up of six months. An age- and sex-matched control group of 43 healthy subjects was recruited for comparison. All patients were pseudophakic at the time of assessment. Retinal sensitivity was assessed using the MP-3 microperimeter (Nidek Technologies, Italy) with a 44-point stimulus grid (Goldmann size III, 4 − 2 staircase strategy) distributed across the central 20°, designed to match the nine-sector ETDRS grid. Macular structure was evaluated by swept-source OCT (DRI OCT Triton; Topcon, Japan). TMT and GCC thickness (µm) were measured in all nine ETDRS sectors. Spearman correlation coefficients were used to assess topographic structure-function relationships. Multivariate linear regression was performed to identify independent predictors of postoperative foveal RS and mean RS.

**Results:**

All 40 MH eyes achieved anatomic closure. Best-corrected visual acuity (BCVA) improved significantly postoperatively (0.94 ± 0.22 to 0.54 ± 0.26 logMAR; *p* < 0.001) but remained inferior to controls (0.12 ± 0.09 logMAR; *p* < 0.001). Mean RS was significantly lower in patients than in controls (24.78 ± 2.73 vs. 26.46 ± 1.77 dB; *p* = 0.002; AUC = 0.721), with significant reductions observed at the fovea and in six of eight parafoveal/perifoveal sectors. The temporal outer sector showed the highest discriminative capacity (AUC = 0.774). Mean GCC thickness (µm) was significantly reduced postoperatively compared to both preoperative values (107.55 ± 11.80 vs. 95.97 ± 13.31 μm; *p* < 0.001) and controls (104.26 ± 7.95 μm; *p* = 0.001). Significant positive topographic correlations were found between RS and TMT at the fovea (ρ = 0.542; *p* < 0.001) and in all four outer ETDRS sectors (ρ ranging from 0.546 to 0.645; all *p* < 0.001), as well as between RS and GCC thickness in multiple sectors (ρ ranging from 0.240 to 0.391; *p* < 0.05). In the multivariate model, specific regional GCC and TMT sector thicknesses were independent predictors of postoperative foveal RS, while preoperative BCVA and MH morphometric parameters showed limited independent predictive value.

**Conclusions:**

Despite successful anatomic MH closure and significant BCVA improvement, retinal sensitivity remained significantly reduced across most extrafoveal ETDRS macular sectors at six months postoperatively. These functional deficits were accompanied by significant GCC thinning in sectors anatomically remote from the macular hole and structurally intact preoperatively. The spatial distribution of these changes is incompatible with damage attributable to the macular hole itself and strongly suggests a causal role for the mechanical trauma of ILM peeling. These findings support the integration of microperimetry as a complementary functional outcome measure in the postoperative assessment of macular hole surgery.

**Supplementary Information:**

The online version contains supplementary material available at https://doi.org/10.1186/s40942-026-00904-4.

## Introduction

Internal limiting membrane (ILM) peeling is a well-established and essential step in the surgical management of macular holes (MH), having consistently demonstrated a positive impact on anatomic closure rates [1–3]. In eyes with MH, the release of tangential tractional forces acting upon the retinal surface in the macular area is believed to facilitate hole closure, with reported success rates exceeding 95% in most contemporary series [1, 4, 5]. However, achieving anatomic closure necessarily requires the removal of the ILM from otherwise healthy perifoveal retina, raising important questions regarding the functional consequences of this maneuver.

The ILM represents the basement membrane of the Müller cells and lies on the innermost surface of the retina, directly adherent to the retinal nerve fiber layer in the macular region.6 Structural changes of the inner retina following ILM peeling have been extensively reported in the literature and are well documented on optical coherence tomography (OCT) [6–8]. These alterations — which include the dissociated optic nerve fiber layer (DONFL) appearance and thinning of the inner retinal layers — appear to occur independently of the adjuvant dye employed and are therefore regarded as a direct mechanical consequence of membrane removal rather than a dye-related toxic effect [9, 10]. Importantly, such structural changes have been associated with reduced retinal sensitivity and the development of microscotomas, which may carry meaningful implications for the functional visual outcomes of affected patients [10, 11]. The functional implications of these structural changes, however, have yet to be fully established and continue to be debated [8–11]. 

Despite this, the functional impact of ILM peeling is frequently overlooked in routine clinical practice, largely because the anatomic success of MH surgery is accompanied by substantial gains in visual acuity (VA) — the most widely used metric for functional assessment in the pre- and postoperative evaluation of these patients. Nevertheless, VA is an inherently limited endpoint, reflecting only foveal resolving capacity under optimal testing conditions, and is therefore insufficient to capture the full spectrum of functional changes occurring across the broader macular area following surgery. A more thorough functional evaluation, encompassing retinal sensitivity assessment throughout the macular region, is warranted to comprehensively characterize the visual outcomes associated with ILM peeling.

In this context, microperimetry offers a uniquely suited approach. By combining differential light sensitivity testing with simultaneous real-time fundus imaging and an integrated eye-tracking system, microperimetry enables precise, stimulus-by-stimulus delivery to predefined retinal loci — independent of fixation instability [12, 13]. This design allows for direct point-by-point topographic correlation between functional sensitivity data and the underlying retinal structure, providing a level of structure-function analysis that conventional perimetry cannot achieve [14]. Prior microperimetry studies in MH surgery have demonstrated postoperative reductions in mean retinal sensitivity (Tadayoni et al. 2012; Lai et al. 2019; Sborgia et al. 2020) and associations between fixation stability and anatomic outcomes (Imai et al. 2018); structure-function correlations using OCT and microperimetry have also been reported, largely using global or foveal OCT parameters (Inoue et al. 2022). The present study extends this literature by employing a 44-point MP-3 grid explicitly matched to all nine ETDRS sectors simultaneously — enabling direct sector-by-sector anatomical correspondence between RS and concurrent OCT measurements of both TMT and GCC — and by applying a multivariate regression framework to identify independent sector-level OCT predictors of postoperative foveal RS [12, 15–17].

Therefore, the purpose of the present study was to evaluate retinal sensitivity (RS) assessed by microperimetry in the macular region of patients undergoing surgical repair of MH, and to investigate the topographic correlation between RS and both total macular thickness (TMT) and ganglion cell complex (GCC) thickness obtained by OCT — establishing point-by-point anatomic correspondence between sensitivity measurements and their structurally matched retinal areas — at six months following successful MH surgery with ILM peeling.

## Methods

### Study design

This was an observational, analytical, cross-sectional study including patients who underwent pars plana vitrectomy (PPV) with internal limiting membrane (ILM) peeling for idiopathic macular hole (MH) repair, with a minimum postoperative follow-up of six months. An age- and sex-matched control group of healthy subjects was recruited for structural and functional comparisons.

### Participants

Consecutive patients who underwent MH surgery at the Juiz de Fora Eye Hospital (HOJF), Juiz de Fora, Minas Gerais, Brazil, between 2022 and 2024 were enrolled. Written informed consent was obtained from all participants. The study was approved by the Research Ethics Committee of the Federal University of Juiz de Fora (UFJF; protocol No. 5.510.781; CAAE: 55455922.1.0000.5147) and conducted in accordance with the tenets of the Declaration of Helsinki.

### Inclusion criteria

Patients were eligible if they had undergone PPV with ILM peeling for idiopathic MH at least six months prior to enrollment and were pseudophakic at the time of functional and structural assessment — either from prior uncomplicated cataract surgery or from combined phacoemulsification performed at the time of MH repair. This criterion was adopted to eliminate the confounding effects of lens opacity on microperimetry results. Additional inclusion criteria were: age between 50 and 75 years; refractive error within ± 5.0 diopters (D) spherical and ± 3.0 D cylindrical; preoperative best-corrected visual acuity (BCVA) between 20/25 and 20/200; preoperative intraocular pressure (IOP) ≤ 21 mmHg; and adequate cooperation for OCT and microperimetry examination.

Control subjects were recruited from patients attending routine or refractive outpatient appointments, including accompanying relatives. Inclusion criteria for controls were: age within ± 5 years of the matched MH patient; BCVA of 20/20; refractive error within ± 5.0 D spherical and ± 3.0 D cylindrical; IOP < 21 mmHg; normal optic nerve and macular appearance on biomicroscopy; no prior ocular disease (except uncomplicated cataract surgery performed more than six months prior); no systemic conditions including diabetes mellitus, decompensated hypertension, congestive heart failure, stroke, or renal failure; and adequate cooperation for OCT and microperimetry examination.

### Exclusion criteria

Patients were excluded if they had: secondary MH; intra- or postoperative complications (retinal detachment, choroidal detachment, vitreous or preretinal hemorrhage, elevated IOP, endophthalmitis, silicone oil tamponade, posterior capsule rupture, or scleral-fixated IOL); prior rhegmatogenous retinal detachment; complicated cataract surgery; prior intravitreal anti-VEGF or corticosteroid injections; prior PPV; pre-existing ocular disease (glaucoma, optic neuropathy, diabetic retinopathy, vascular occlusion, age-related macular degeneration, or MH associated with epiretinal membrane); axial length > 25 mm; MH requiring non-standard surgical technique; phakic lens status at the time of examination; or significant media opacity.

### Surgical technique

All procedures were performed by a single surgeon (LPC) at HOJF using 25-gauge valved trocar PPV (Constellation Vision System; Alcon Laboratories, Fort Worth, TX, USA), either as a standalone procedure or combined with phacoemulsification and intraocular lens implantation. Trocars were placed 3.5 mm from the limbus. Triamcinolone acetonide was used to enhance vitreous visualization. Posterior vitreous detachment was induced when absent. The ILM was stained with MembraneBlue-Dual (DORC International, Zuidland, The Netherlands) and peeled using a 25-gauge Eckardt end-gripping forceps using the pinch-and-peel technique, over an area of at least 3 disc diameters (DD) centered on the fovea, encompassing the entire macular region within the temporal vascular arcades. This extent was intentionally chosen to ensure anatomical correspondence between the surgically treated area and the 6-mm diameter ETDRS grid used for both OCT thickness mapping and microperimetry point distribution. After peripheral vitreous base revision, fluid-air exchange was performed, followed by endotamponade with 20% sulfur hexafluoride (SF6) gas. No conjunctival or scleral sutures were placed. Macular hole closure was confirmed by postoperative OCT.

### Optical coherence tomography

Structural macular assessment was performed using a swept-source OCT device (DRI OCT Triton; Topcon Corporation, Tokyo, Japan), operating at a wavelength of 1050 nm, with axial resolution of approximately 2 μm and lateral resolution ≤ 20 μm. A standardized 3D macular acquisition protocol covering a 7 × 7 mm area centered on the fovea was used (256 × 512 density; ~2 s acquisition at 100,000 A-scans/second), supplemented by high-resolution B-scan cross-sections (up to 4096 pixels) and volumetric reconstructions. Images were acquired under pharmacological mydriasis. Only scans with a quality index ≥ 60 were accepted for analysis.

Total macular thickness (TMT, µm) and ganglion cell complex (GCC) thickness (µm) were automatically segmented by the device software and reported according to the nine-sector ETDRS grid (6 mm diameter, fovea-centered): one central sector, four inner parafoveal sectors (temporal, superior, nasal, and inferior), and four outer perifoveal sectors.

All automated segmentation outputs were reviewed by two independent masked graders (RSP and GCSB). Scans exhibiting boundary errors at the ILM, inner plexiform layer, or ganglion cell layer interface — particularly in sectors affected by DONFL artifacts or residual foveal contour irregularity following hole closure — were manually corrected using the device’s integrated boundary adjustment tool prior to data export. Scans for which satisfactory segmentation could not be achieved were excluded from the analysis. The DRI OCT Triton platform has demonstrated high intersession reproducibility for TMT and GCC thickness measurements in published validation studies, with intraclass correlation coefficients exceeding 0.95 for ETDRS sector-level values. In preoperative scans, MH morphometric parameters were also measured, including minimum linear diameter (MLD), base diameter (BD) at the level of the retinal pigment epithelium (RPE), and hole height at the nasal and temporal margins.

OCT and microperimetry examinations were performed at six months postoperatively, preferably in the same session or within a maximum interval of one week.

### Microperimetry

**Fig. 1:**
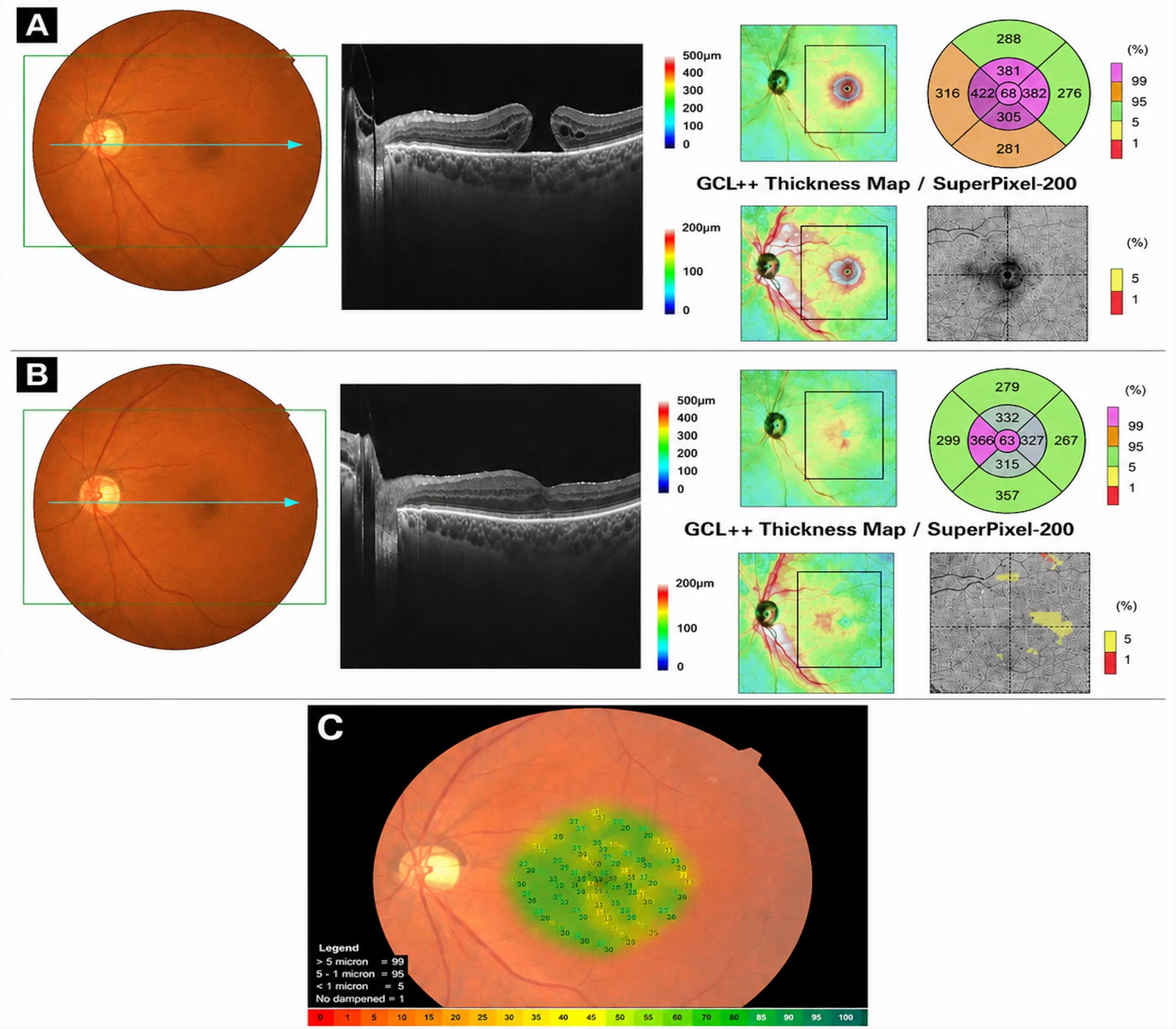
Representative example of multimodal structural and functional macular assessment in a patient with idiopathic macular hole (MH). (**A**) Preoperative findings: color fundus photography with OCT B-scan confirming full-thickness MH, TMT and GCC maps displayed according to the nine-sector ETDRS grid. (**B**) Postoperative findings at six months: OCT B-scan demonstrating successful anatomic closure, with corresponding TMT and GCC measurements. (**C**) MP-3 microperimetry map at six months. Green dots indicate retinal sensitivity within normal limits; yellow dots represent loci with mildly reduced sensitivity

**Fig. 2:**
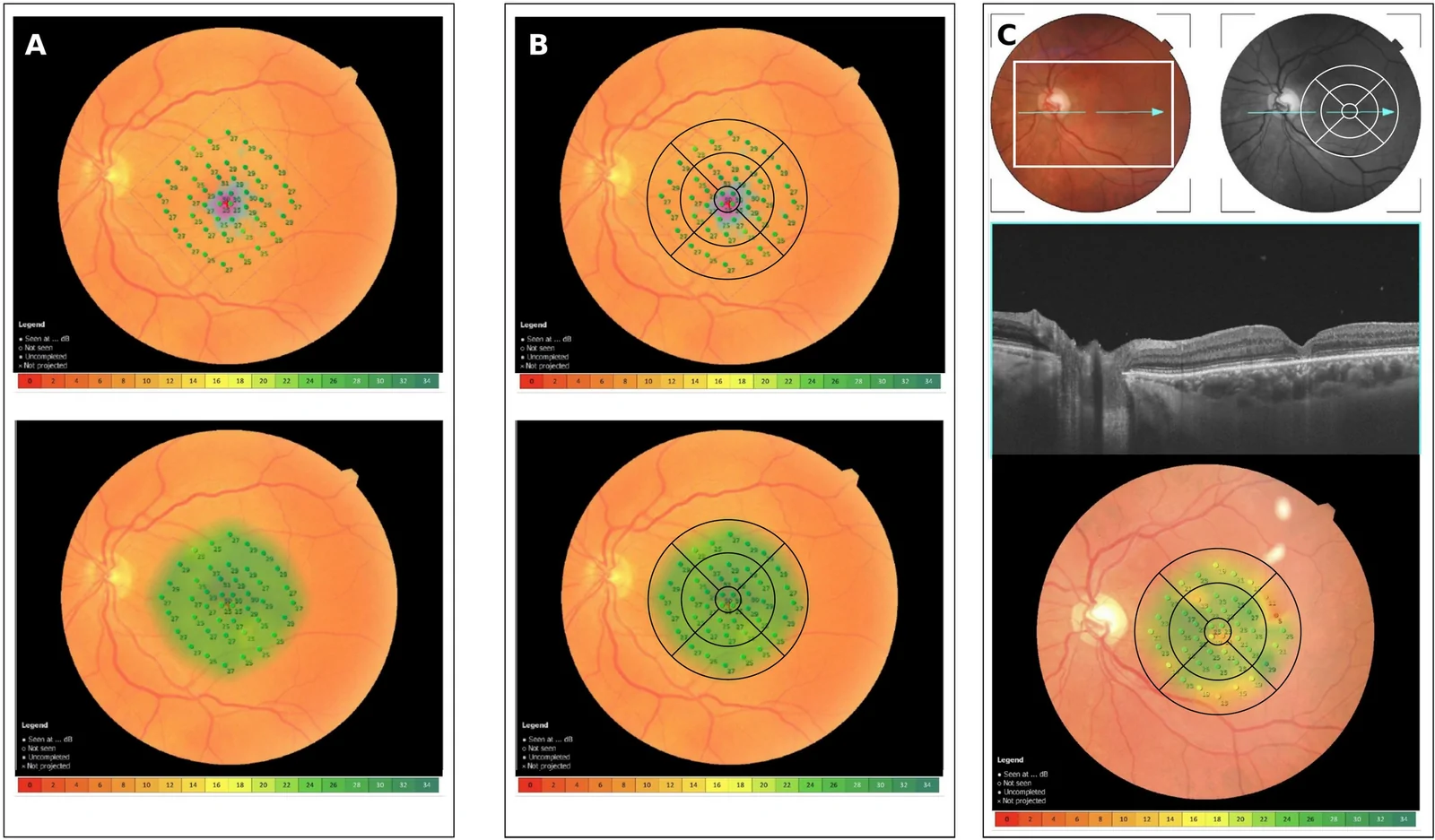
Schematic representation of color fundus photography, microperimetry, and OCT images demonstrating the topographic correlation between microperimetry testing points and the ETDRS grid map. (**A**) Color fundus photograph combined with microperimetry showing the 44 tested loci. (**B**) Same images with the ETDRS grid map overlaid onto the microperimetry examination. (**C**) Representative example from a study participant showing the postoperative OCT B-scan with closed macular hole and the correlation between areas tested by microperimetry and the superimposed ETDRS grid map

Macular retinal sensitivity (RS) was assessed using the MP-3 microperimeter (Nidek Technologies, Padova, Italy). A 44-point stimulus grid covering the central 20° of the visual field (corresponding to approximately ± 10° from the fovea) was used, with stimuli distributed to match the nine sectors of the ETDRS grid — five stimulus points per inner and outer sector, and four points within the central 1 mm diameter circle — thereby enabling direct point-by-point topographic correlation between RS and OCT thickness measurements (Figs. 1 and 2).

Stimuli corresponded to a Goldmann size III target (200 ms duration) presented on a white background (luminance: 1.27 cd/m²; 4 apostilbs). Retinal sensitivity thresholds were determined using a 4 − 2 full-threshold staircase strategy, expressed in decibels (dB) on a scale from 0 dB (maximum stimulus luminance) to 34 dB (minimum luminance). Areas unable to detect the maximum stimulus intensity were classified as absolute scotomas (0 dB). Mean RS was calculated as the arithmetic mean of all 44 threshold values.

An integrated real-time eye-tracking system ensured precise stimulus delivery to predefined retinal loci, compensating for involuntary eye movements throughout the examination. All tests were performed under pharmacological mydriasis with the fellow eye occluded. To minimize learning effects, testing was initiated in the fellow (non-operated) eye, followed by two practice runs prior to the definitive examination of the study eye, a 15-minute rest period, and a 5-minute mesopic adaptation phase. A red cross fixation target (1° diameter) was used throughout testing.

Only examinations with false-positive and false-negative rates below 33% were included in the analysis, consistent with the reliability criterion adopted in published MP-3 studies in macular disease populations, including prior microperimetry studies in macular hole surgery [11, 12]. This threshold reflects the different reliability framework of the MP-3 compared with conventional automated perimetry: the integrated real-time fundus tracking system continuously repositions each stimulus to the predefined retinal locus regardless of fixation instability, maintaining topographic accuracy of stimulus delivery independently of fixation quality. The false-positive and false-negative rates in MP-3 testing therefore primarily reflect inattention or fatigue rather than spatial positional error.

### Statistical analysis

Continuous variables were tested for normality using the Shapiro-Wilk test. Comparisons between MH eyes and matched controls were performed using parametric or non-parametric tests as appropriate. Topographic correlations between structural parameters (TMT and GCC thickness) and functional measurements (RS) across ETDRS sectors were assessed using Pearson or Spearman correlation coefficients, according to the distribution of each variable. To address multiple comparisons across sectoral analyses, the Benjamini-Hochberg false discovery rate (FDR) correction was applied within each comparison family (retinal sensitivity sectors; TMT sectors; GCC sectors), with significance defined as FDR-corrected q < 0.10. Univariate and multivariate **linear** regression analyses were performed with postoperative foveal RS and mean RS as dependent variables, and pre- and postoperative BCVA and OCT morphometric parameters as independent variables. Variance inflation factors (VIF) were inspected for all predictors included in the multivariate models; no VIF exceeded 5.0. Given the modest sample size (*n* = 40), regression findings should be considered exploratory and hypothesis-generating. Statistical significance for non-multiplicity-adjusted analyses was set at *p* < 0.05. All analyses were performed using SPSS software, version 21 (IBM Corp., Armonk, NY, USA).

## Results

Demographic and clinical characteristics of the study groups are summarized in Table 1. No statistically significant differences were found between MH patients and controls regarding mean age (*p* = 0.056) or sex distribution (*p* = 0.292), confirming adequate group matching. Patients exhibited significantly worse BCVA compared to controls both preoperatively (0.94 ± 0.22 vs. 0.12 ± 0.09 logMAR; *p* < 0.001) and postoperatively (0.54 ± 0.26 vs. 0.12 ± 0.09 logMAR; *p* < 0.001). A significant improvement in BCVA was observed in the patient group between the pre- and postoperative assessments (*p* < 0.001). No significant difference was found between groups in intraocular pressure (*p* = 0.43).

**Table 1:** Demographic and clinical characteristics of the patient and control groups, including age, sex, pre- and postoperative best-corrected visual acuity (BCVA, logMAR), and intraocular pressure (IOP). Continuous data are expressed as mean ± standard deviation

Variable	Patients (*n* = 40)	Controls (*n* = 43)	*p*-value
Age (years)	66.85 ± 8.23	63.79 ± 7.74	0.056*
Sex, male	17	13	0.292**
Sex, female	23	30	
Preoperative BCVA (logMAR)	0.94 ± 0.22	0.12 ± 0.09	< 0.001*
Postoperative BCVA (logMAR)	0.54 ± 0.26	0.12 ± 0.09	< 0.001*
Pre vs. post BCVA (patients)	—	—	< 0.001*
IOP (mmHg)	13.93 ± 2.49	13.48 ± 2.67	0.43*

*Unpaired Student’s t-test. **Chi-square test. BCVA: best-corrected visual acuity; IOP: intraocular pressure; mmHg: millimeters of mercury. Bold values indicate *p* < 0.05

Retinal sensitivity (RS) values assessed by microperimetry are presented in Table 2. MH patients demonstrated significantly reduced RS compared to controls in multiple macular sectors. Statistically significant differences were observed at the fovea (24.26 ± 3.80 vs. 27.07 ± 2.84 dB; *p* = 0.0003) and in the TI, SI, and II sectors (p ranging from 0.0014 to 0.0098). Among the outer sectors, significant differences were identified in TO and SO, with the TO sector exhibiting the highest discriminative capacity between groups (AUC = 0.774). Mean RS was significantly lower in patients than in controls (24.78 ± 2.73 vs. 26.46 ± 1.77 dB; *p* = 0.0016; AUC = 0.721). No significant differences were found in the NI, NO, or IO sectors, suggesting a preferential pattern of functional impairment in the juxtafoveal and temporal macular regions. Primary findings were maintained after Benjamini-Hochberg FDR correction (q < 0.10).

MH size distribution: medium holes (> 250 and ≤ 400 μm) were the most common, accounting for 20 cases (50.0%). Large holes (> 400 and ≤ 550 μm) were found in 9 cases (22.5%), small holes (< 250 μm) in 6 cases (15.0%), and extra-large holes (> 550 and ≤ 800 μm) in 5 cases (12.5%). No extra-extra-large or giant holes were identified. MH morphometric parameters assessed by OCT showed a mean MLD of 501.22 ± 176.65 μm, mean base diameter of 1127 ± 342.64 μm, mean nasal height of 294.47 ± 150.87 μm, mean temporal height of 282.81 ± 146.63 μm, and total height of 577.28 ± 158.32 μm (Supplemental Fig. 1).

**Table 2:** Comparison of retinal sensitivity values assessed by microperimetry between patients with macular hole and controls, by macular sector. Data are expressed as mean ± standard deviation (dB)

Sector	Patients (*n* = 40)	Controls (*n* = 43)	*p*-value	AUC
Fovea	24.26 ± 3.80	27.07 ± 2.84	**0.0003**	**0.747**
TI	25.34 ± 3.90	27.57 ± 1.55	**0.0014**	**0.756**
SI	24.89 ± 4.10	26.77 ± 1.76	**0.0098**	**0.678**
NI	25.98 ± 3.04	26.92 ± 1.96	0.1010	0.579
II	25.19 ± 2.40	26.51 ± 1.98	**0.0082**	**0.670**
TO	23.93 ± 3.37	26.72 ± 1.83	**< 0.001**	**0.774**
SO	24.48 ± 2.42	25.87 ± 2.16	**0.0073**	**0.672**
NO	25.43 ± 2.82	25.99 ± 2.14	0.3223	0.571
IO	24.31 ± 3.09	25.38 ± 2.28	0.0794	0.610
**Mean**	**24.78 ± 2.73**	**26.46 ± 1.77**	**0.0016**	**0.721**

AUC: area under the ROC curve; TI: temporal inner; SI: superior inner; NI: nasal inner; II: inferior inner; TO: temporal outer; SO: superior outer; NO: nasal outer; IO: inferior outer. Bold values indicate *p* < 0.05

**Fig. 3:**
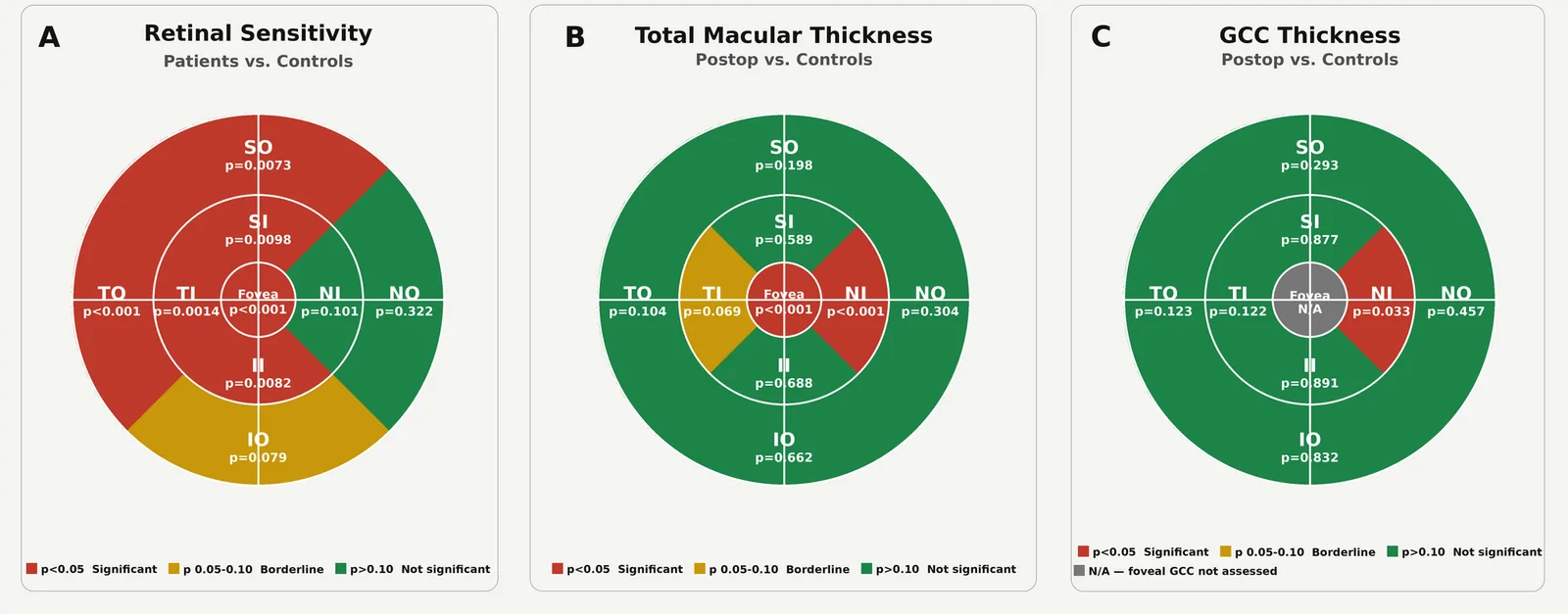
Color-coded ETDRS macular sector maps illustrating the statistical significance of between-group differences in retinal sensitivity, total macular thickness, and ganglion cell complex thickness. Each sector is colored according to the p-value: red = statistically significant (*p* < 0.05); yellow = borderline (p 0.05–0.10); green = not significant (*p* > 0.10). (**A**) Retinal sensitivity (patients vs. controls). (**B**) Total macular thickness (postoperative vs. controls). (**C**) Ganglion cell complex thickness (postoperative vs. controls). TI: temporal inner; SI: superior inner; NI: nasal inner; II: inferior inner; TO: temporal outer; SO: superior outer; NO: nasal outer; IO: inferior outer

**Fig. 4:**
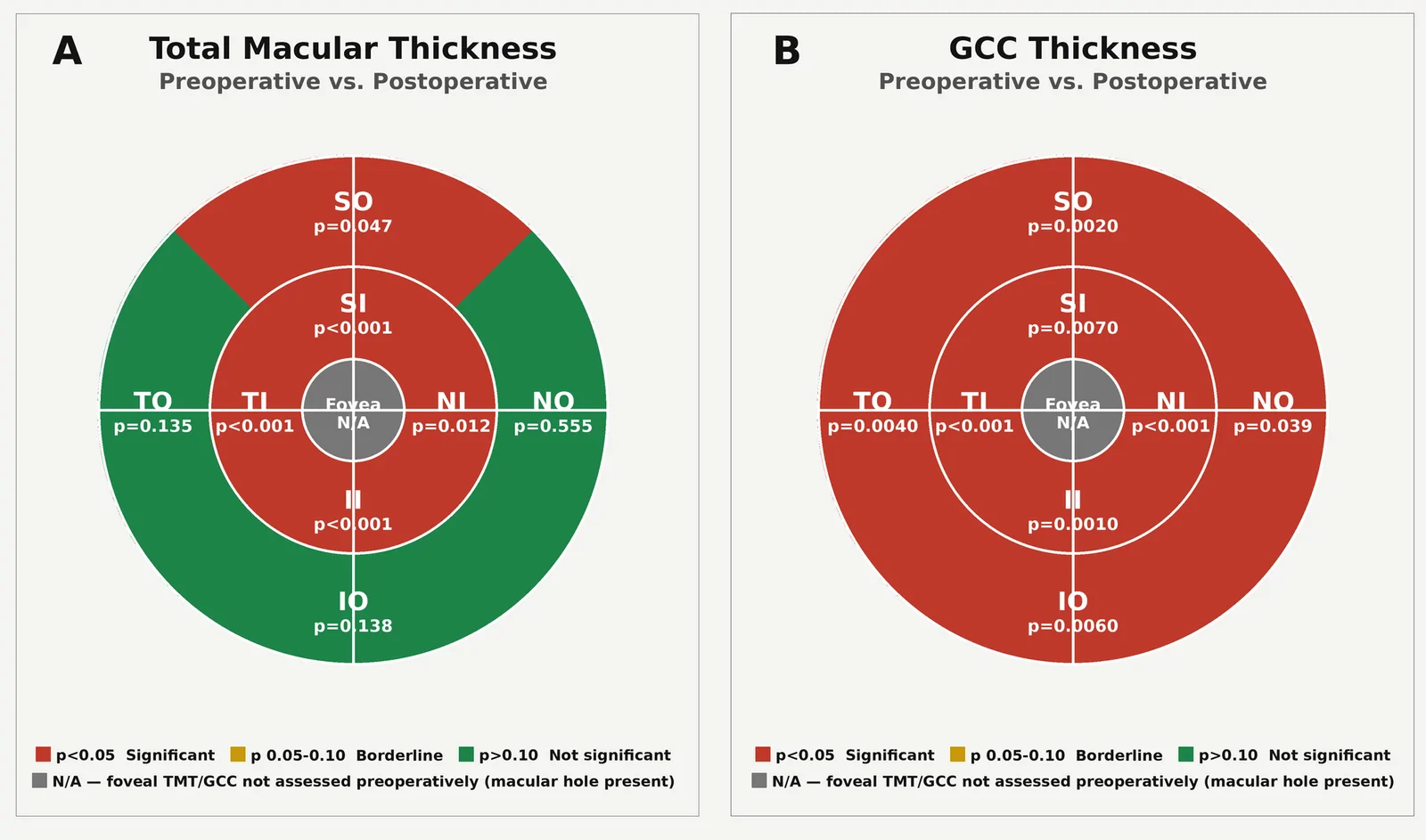
Color-coded ETDRS macular sector maps comparing preoperative and postoperative structural measurements within the macular hole patient group. Each sector is colored according to the p-value of the preoperative vs. postoperative comparison (same color scheme as Fig. 3). The foveal sector (gray) was not assessable preoperatively. (**A**) Total macular thickness (TMT): significant postoperative reductions in TI, SI, NI, II, and SO sectors. (**B**) Ganglion cell complex (GCC) thickness: significant postoperative reductions in all eight assessable ETDRS sectors

Total macular thickness (TMT, µm) measurements obtained by OCT are presented in Table 3. Significant reductions in macular thickness were observed postoperatively compared to preoperative values in the TI, SI, NI, II, and SO sectors, as well as in mean global macular thickness (p ranging from < 0.001 to 0.047). When comparing postoperative patients with controls, most sectors showed no statistically significant differences; however, foveal and NI thicknesses were significantly greater in patients postoperatively (Figs. 3 and 4).

**Table 3:** Comparison of total macular thickness (TMT, µm) assessed by OCT in MH patients (preoperative and postoperative) and in controls. Data are expressed as mean ± standard deviation

Sector	Pre (*n* = 40)	Post (*n* = 40)	Control (*n* = 43)	*p* Pre vs. Post	*p* Pre vs. Ctrl	*p* Post vs. Ctrl	AUC Pre vs. Ctrl	AUC Post vs. Ctrl
Fovea	N/A	272.10 ± 44.12	241.26 ± 34.75	N/A	N/A	**0.0009**	N/A	0.761
TI	331.90 ± 51.24	286.21 ± 36.46	298.00 ± 16.32	**0.0001**	**0.0003**	0.069	0.686	0.323
SI	339.92 ± 46.31	307.36 ± 23.66	309.88 ± 17.36	**0.0002**	**0.0004**	0.589	0.708	0.467
NI	357.03 ± 49.98	333.62 ± 25.94	310.67 ± 18.13	**0.012**	**0.0001**	**0.0001**	0.816	0.787
II	348.49 ± 50.11	310.13 ± 20.36	308.29 ± 20.75	**0.0001**	**0.0001**	0.688	0.763	0.548
TO	255.49 ± 23.81	247.21 ± 24.62	254.36 ± 11.60	0.135	0.790	0.104	0.465	0.338
SO	274.26 ± 23.26	263.92 ± 21.83	269.36 ± 14.80	**0.047**	0.266	0.198	0.544	0.380
NO	292.03 ± 20.95	289.00 ± 24.03	284.31 ± 15.40	0.555	0.065	0.304	0.596	0.505
IO	265.49 ± 23.72	257.95 ± 20.58	259.67 ± 13.64	0.138	0.185	0.662	0.540	0.419
**Mean**	**288.69 ± 26.54**	**274.70 ± 20.39**	**275.05 ± 13.49**	**0.011**	**0.006**	**0.927**	**0.658**	**0.449**

AUC: area under the ROC curve; N/A: not applicable (foveal sector could not be measured preoperatively due to the macular hole); TI: temporal inner; SI: superior inner; NI: nasal inner; II: inferior inner; TO: temporal outer; SO: superior outer; NO: nasal outer; IO: inferior outer. Bold values indicate *p* < 0.05

Ganglion cell complex (GCC) thickness (µm) values are shown in Table 4. Significant reductions were observed in all sectors from the preoperative to the postoperative period (p ranging from < 0.001 to 0.039). Mean GCC thickness decreased significantly from 107.55 ± 11.80 μm preoperatively to 95.97 ± 13.31 μm postoperatively (*p* < 0.001), remaining significantly lower than control values (104.26 ± 7.95 μm; *p* = 0.001). Notably, the NI sector showed significantly increased GCC thickness in patients postoperatively compared to controls (*p* = 0.033), a finding also observed for TMT in the same sector (Figs. 3 and 4).

**Table 4:** Comparison of ganglion cell complex (GCC) thickness (µm) assessed by OCT in MH patients (preoperative and postoperative) and in controls. Data are expressed as mean ± standard deviation

Sector	Pre (*n* = 40)	Post (*n* = 40)	Control (*n* = 43)	*p* Pre vs. Post	*p* Pre vs. Ctrl	*p* Post vs. Ctrl	AUC Pre vs. Ctrl	AUC Post vs. Ctrl
TI	134.03 ± 38.93	98.50 ± 32.56	106.79 ± 6.73	**< 0.001**	**0.0001**	0.122	0.848	0.155
SI	141.85 ± 39.50	118.92 ± 33.82	118.07 ± 7.45	**0.007**	**0.001**	0.877	0.824	0.389
NI	159.62 ± 40.45	127.05 ± 38.41	113.36 ± 8.12	**0.0004**	**< 0.001**	**0.033**	0.952	0.687
II	147.72 ± 38.90	118.88 ± 34.33	118.10 ± 10.72	**0.001**	**< 0.001**	0.891	0.857	0.390
TO	103.67 ± 29.63	84.45 ± 27.88	91.67 ± 8.47	**0.004**	**0.017**	0.123	0.760	0.232
SO	120.03 ± 30.60	97.67 ± 31.04	103.10 ± 8.98	**0.002**	**0.002**	0.293	0.810	0.288
NO	135.32 ± 29.17	121.60 ± 29.29	117.93 ± 10.31	**0.039**	**0.001**	0.457	0.826	0.490
IO	120.70 ± 29.04	102.50 ± 28.33	103.52 ± 11.00	**0.006**	**0.001**	0.832	0.826	0.383
**Mean**	**107.55 ± 11.80**	**95.97 ± 13.31**	**104.26 ± 7.95**	**0.0001**	**0.145**	**0.001**	**0.603**	**0.259**

AUC: area under the ROC curve; TI: temporal inner; SI: superior inner; NI: nasal inner; II: inferior inner; TO: temporal outer; SO: superior outer; NO: nasal outer; IO: inferior outer. Bold values indicate *p* < 0.05

Correlations between BCVA and postoperative microperimetry values are presented in Table 5. Preoperative BCVA showed significant inverse correlations with postoperative RS in the SI (*r* = − 0.394; *p* = 0.012), II (*r* = − 0.553; *p* = 0.0002) sectors, and mean RS (*r* = − 0.405; *p* = 0.010). Postoperative BCVA was significantly inversely correlated with RS in the NI (*r* = − 0.353; *p* = 0.025), II (*r* = − 0.434; *p* = 0.005), SO (*r* = − 0.335; *p* = 0.035), and IO (*r* = − 0.312; *p* = 0.050) sectors. These findings suggest that both pre- and postoperative BCVA influence postoperative macular functional recovery, particularly in the inner macular sectors.

Analysis of correlations between postoperative RS and MH morphometric parameters revealed a positive unadjusted correlation between mean RS and MLD (*r* = 0.343; *p* = 0.040); however, this association did not survive Benjamini-Hochberg FDR correction. Stratified analysis by MH size category showed no significant difference in mean RS across size groups (Kruskal-Wallis, *p* > 0.05). No significant correlations were found between postoperative RS and base diameter, nasal height, temporal height, or total height for any individual sector. No significant correlations were found between any MH dimension and postoperative BCVA.

**Table 5:** Correlations between preoperative and postoperative BCVA and postoperative retinal sensitivity (RS) assessed by microperimetry, by macular sector

Sector	Preop BCVA (*r*)	*p*	Postop BCVA (*r*)	*p*
Fovea	-0.247	0.125	-0.251	0.119
TI	-0.200	0.217	-0.162	0.318
SI	**-0.394**	**0.012**	-0.251	0.118
NI	-0.189	0.242	**-0.353**	**0.025**
II	**-0.553**	**0.0002**	**-0.434**	**0.005**
TO	-0.118	0.470	-0.061	0.707
SO	-0.252	0.116	**-0.335**	**0.035**
NO	-0.256	0.112	-0.233	0.149
IO	-0.236	0.143	**-0.312**	**0.050**
**Mean**	**-0.405**	**0.010**	**-0.306**	**0.055**

r: Pearson or Spearman correlation coefficient; BCVA: best-corrected visual acuity; TI: temporal inner; SI: superior inner; NI: nasal inner; II: inferior inner; TO: temporal outer; SO: superior outer; NO: nasal outer; IO: inferior outer. Bold values indicate *p* < 0.05

**Fig. 5:**
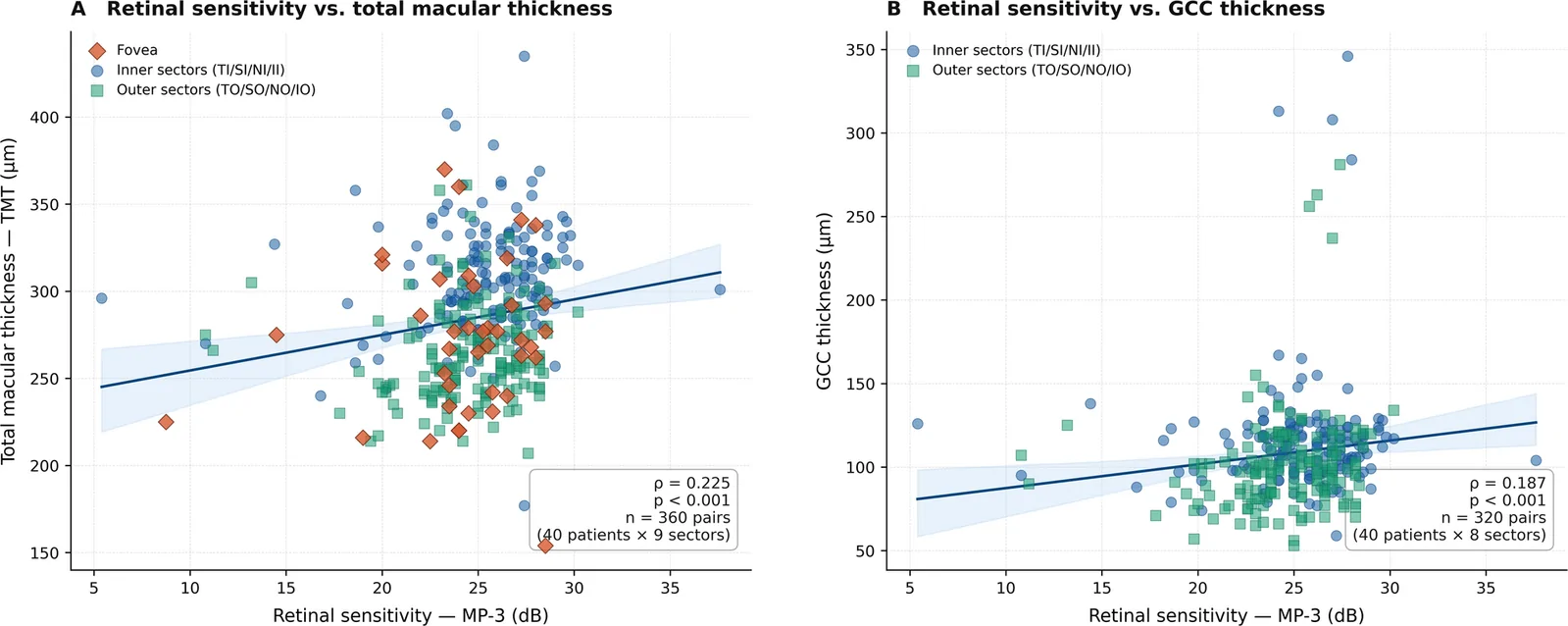
Scatter plots illustrating the topographic structure-function relationship between postoperative retinal sensitivity (RS) assessed by microperimetry (MP-3) and postoperative macular structural parameters measured by optical coherence tomography (OCT), using sector-level data from all 40 macular hole patients at six months postoperatively. Each data point represents a single sector-patient pair. Points are color-coded by macular region: blue circles = inner parafoveal sectors; green squares = outer perifoveal sectors; orange diamonds = foveal sector (panel A). The solid line and shaded area are provided for visual reference only and do not constitute formal statistical estimates. *These scatter plots are provided for illustrative purposes only* to depict the overall directional trend across sector-patient pairs; they are not the primary statistical analyses of this paper (see Table 6 for the sector-specific Spearman correlations, each computed independently within a single ETDRS sector across the 40 patients). The pooled ρ values displayed (TMT: ρ = 0.225; GCC: ρ = 0.187) should be interpreted with caution as they do not account for within-eye clustering of sectors. (**A**) RS vs. total macular thickness (TMT): ρ = 0.225; *n* = 360 sector-patient pairs. (**B**) RS vs. ganglion cell complex (GCC) thickness: ρ = 0.187; *n* = 320 sector-patient pairs

Structure-function correlations between postoperative retinal sensitivity and postoperative OCT thickness measurements are presented in Table 6. Significant correlations between RS and TMT (µm) were found at the fovea (ρ = 0.542; *p* = 0.0003), NI (ρ = 0.339; *p* = 0.033), TO (ρ = 0.645; *p* < 0.001), NO (ρ = 0.632; *p* < 0.001), SO (ρ = 0.628; *p* < 0.001), IO (ρ = 0.546; *p* = 0.0003), and mean (ρ = 0.503; *p* = 0.001). The strongest correlations were observed in the outer sectors. For GCC thickness (µm), significant correlations were found in TI (ρ = 0.391; *p* = 0.0003), TO (ρ = 0.369; *p* = 0.001), SO (ρ = 0.375; *p* = 0.001), IO (ρ = 0.240; *p* = 0.029), and mean (ρ = 0.320; *p* = 0.003). Overall, structure-function relationships were more consistent for TMT than for GCC, particularly at the fovea and outer perifoveal sectors (Figs. 3, 4 and 5).

**Table 6:** Spearman correlation coefficients (ρ) between postoperative retinal sensitivity assessed by microperimetry and postoperative total macular thickness (TMT, µm) and ganglion cell complex (GCC) thickness (µm), by ETDRS macular sector

Sector	TMT ρ	*p*	GCC ρ	*p*
Fovea	**0.542**	**0.0003**	*	*
TI	0.256	0.111	**0.391**	**0.0003**
NI	**0.339**	**0.033**	-0.152	0.169
SI	0.119	0.463	0.118	0.287
II	0.176	0.277	0.180	0.104
TO	**0.645**	**< 0.001**	**0.369**	**0.0006**
NO	**0.632**	**< 0.001**	0.151	0.172
SO	**0.628**	**< 0.001**	**0.375**	**0.0005**
IO	**0.546**	**0.0003**	**0.240**	**0.029**
**Mean**	**0.503**	**0.0009**	**0.320**	**0.003**

ρ: Spearman correlation coefficient; TMT: total macular thickness; GCC: ganglion cell complex; TI: temporal inner; SI: superior inner; NI: nasal inner; II: inferior inner; TO: temporal outer; SO: superior outer; NO: nasal outer; IO: inferior outer. *Foveal GCC was not assessed as the foveal avascular zone lacks ganglion cells. Bold values indicate *p* < 0.05

### Multivariate linear regression analysis

Multivariate linear regression analysis of **postoperative foveal RS** as the dependent variable identified several structural OCT parameters as independent predictors. Among MH morphometric measures, temporal hole height was the only significant predictor of postoperative foveal RS (β = −0.064; *p* = 0.024), indicating that greater temporal height was associated with lower foveal RS. The remaining MH dimensions (MLD, base diameter, nasal height, and total height) did not reach statistical significance (*p* > 0.05). For GCC thickness, significant independent associations with foveal RS were found in the SI (β = −1.471; *p* = 0.002), II (β = 0.550; *p* = 0.032), and SO (β = 0.550; *p* = 0.037) sectors. For TMT, significant independent associations were found in the TI (β = −0.315; *p* = 0.001), II (β = 0.342; *p* = 0.001), and IO (β = −0.680; *p* = 0.001) sectors. Preoperative and postoperative BCVA, mean GCC, and mean TMT were not significant independent predictors (*p* > 0.05).

When **mean postoperative RS** was used as the dependent variable in the multivariate linear regression model, nasal hole height was the only variable — morphometric or structural — that independently predicted mean RS (β = 0.054; *p* = 0.023), suggesting that greater vertical extent of the MH on the nasal side was associated with higher mean postoperative RS. The remaining MH morphometric dimensions (MLD, base diameter, temporal height, total height) did not reach statistical significance (*p* > 0.05). No sector-level GCC thickness parameter showed a statistically significant independent association with mean RS in the multivariate model (*p* > 0.05 for all sectors). Similarly, no sector-level TMT parameter reached statistical significance as an independent predictor of mean RS (*p* > 0.05 for all sectors). Preoperative and postoperative BCVA, global mean GCC, and global mean TMT were also not significant independent predictors (*p* > 0.05 for all). These findings indicate that, among all structural parameters evaluated, only the nasal morphological configuration of the MH exerts an independent influence on global mean postoperative RS, while regional OCT structural measures do not independently predict mean RS after adjustment for other predictors.

## Discussion

The present study investigated retinal sensitivity (RS) assessed by microperimetry and its topographic correlation with total macular thickness (TMT, µm) and ganglion cell complex (GCC) thickness (µm) measured by optical coherence tomography (OCT), six months following successful macular hole (MH) surgery with internal limiting membrane (ILM) peeling. The main findings can be summarized as follows: (1) MH patients demonstrated significantly reduced RS compared to age- and sex-matched controls in the majority of macular sectors assessed, despite anatomically successful hole closure and significant improvement in BCVA; (2) both TMT and GCC thickness (µm) were significantly reduced postoperatively relative to preoperative values; (3) significant positive topographic correlations were found between RS and TMT as well as between RS and GCC thickness across multiple ETDRS sectors; and (4) multivariate regression analysis identified specific regional OCT parameters as independent predictors of postoperative foveal RS and mean RS. Collectively, these findings indicate that anatomic success after MH surgery does not translate into complete functional recovery and support the use of microperimetry as a sensitive tool for detecting residual macular dysfunction that escapes conventional visual acuity assessment.

Surgical repair of idiopathic MH with ILM peeling resulted in significant improvement of BCVA at six months postoperatively (0.94 ± 0.22 to 0.54 ± 0.26 logMAR; *p* < 0.001), consistent with the high anatomic closure rates and visual gains consistently reported in the literature.1–5 All 40 eyes in the study achieved MH closure, reflecting the well-established efficacy of modern vitreoretinal surgical technique. However, postoperative BCVA remained significantly inferior to that of age- and sex-matched controls (0.54 ± 0.26 vs. 0.12 ± 0.09 logMAR; *p* < 0.001), indicating persistent functional limitation despite anatomic success. These findings are consistent with prior studies demonstrating that, while ILM peeling significantly improves closure rates and visual outcomes, complete restoration of preoperative visual function is not always achieved.6–8.

The discrepancy between BCVA improvement and residual functional deficit is well recognized in the MH literature. Visual acuity reflects only the resolving capacity of the foveal center under optimal photopic conditions and is therefore insufficient to capture the full spectrum of macular functional changes, particularly those affecting the parafoveal and perifoveal regions. Our findings reinforce the notion that BCVA alone represents a limited functional endpoint in the postoperative evaluation of MH patients, and that additional functional testing is required for a comprehensive assessment of visual outcomes [9–11]. Notably, postoperative BCVA was included as a covariate in the multivariate regression models and did not emerge as a significant independent predictor of postoperative RS (*p* > 0.05), while sector-specific OCT structural parameters did — providing further structural evidence for the functional dissociation between BCVA and RS as outcome measures.

The borderline age difference between groups (approximately 3 years; *p* = 0.056) is statistically non-significant and clinically modest. Published normative data for the MP-3 microperimeter indicate an age-related decline in retinal sensitivity of approximately 0.1–0.2 dB per decade in healthy subjects. The observed 3-year difference would therefore predict a maximum theoretical confound of approximately 0.03–0.06 dB — more than 25-fold smaller than the observed mean RS difference of 1.68 dB between groups — making it highly implausible that the borderline age imbalance accounts for the functional differences reported.

One of the principal findings of the present study was the demonstration that RS, assessed by the MP-3 microperimeter, was significantly reduced in MH patients compared to controls in seven of the nine ETDRS sectors evaluated, including the fovea (24.26 ± 3.80 vs. 27.07 ± 2.84 dB; *p* = 0.0003) and multiple parafoveal and perifoveal sectors. Mean RS was also significantly lower in patients (24.78 ± 2.73 vs. 26.46 ± 1.77 dB; *p* = 0.0016). These findings indicate that, despite successful MH closure, a functionally meaningful and topographically diffuse reduction in macular sensitivity persists at six months postoperatively. Critically, these functional deficits were identified predominantly in the extrafoveal sectors — regions that were anatomically intact preoperatively, as evidenced by the absence of structural damage in these areas on preoperative OCT (Tables 3 and 4).

The temporal outer (TO) sector exhibited the highest discriminative power between groups (AUC = 0.774), followed by the temporal inner (TI) and foveal sectors. This preferential involvement of the temporal macular region is consistent with the known nasal displacement of the retina described after MH surgery with ILM peeling, a phenomenon attributed to the release of tractional forces and the centripetal reorganization of perifoveal tissue toward the disc [12, 13]. Sectors along the nasal meridian (NI, NO, IO) did not reach statistical significance, further supporting this topographic pattern of functional compromise.

Prior microperimetry studies in MH surgery have demonstrated postoperative reductions in mean retinal sensitivity (Tadayoni et al. 2012; Lai et al. 2019; Sborgia et al. 2020) and reported correlations between fixation stability and anatomic outcomes (Imai et al. 2018). Structure-function analyses using OCT and microperimetry have also been reported, notably by Inoue et al. (2022), who found significant correlations between RS and macular thickness after MH surgery. The present study extends this literature in three specific respects: (1) we employed a 44-point stimulus grid explicitly matched to all nine ETDRS sectors simultaneously — enabling true point-by-point anatomical correspondence rather than global or foveal-only comparisons; (2) we concurrently evaluated both TMT and GCC as distinct structural correlates of RS, allowing separation of total retinal thickness contributions from those of the inner retinal layers specifically; and (3) we applied a multivariate regression framework to identify independent sector-level OCT predictors of postoperative foveal RS, providing a prognostic analytical framework not previously reported in MH populations [28, 29]. 

In particular, the ROC analysis demonstrated moderate discriminative performance (AUC = 0.721 for mean RS), underscoring the potential utility of microperimetry as a supplementary functional outcome measure in MH surgery.

The observation that NI, NO, and IO sectors were not significantly affected may reflect the redistribution of macular tissue toward the nasal quadrant following surgery, as previously described [12, 13]. This asymmetric pattern of sensitivity loss highlights the importance of sector-specific functional analysis rather than reliance on global mean sensitivity alone.

Total macular thickness (TMT, µm) was significantly reduced postoperatively compared to preoperative values in the TI, SI, NI, II, and SO sectors, as well as in mean global TMT (p ranging from < 0.001 to 0.047). This postoperative thinning is consistent with the resolution of vitreoretinal traction, regression of intraretinal cystoid spaces, and structural reorganization of the foveal architecture following ILM peeling — changes that have been well documented in prior OCT-based studies [18–20]. 

Despite the observed reductions, mean postoperative TMT did not differ significantly from controls (*p* = 0.927), suggesting that, on average, surgery promoted structural normalization of the macular profile rather than diffuse thinning below physiological levels [20, 21]. 

Notably, two sectors showed significantly greater thickness in postoperative patients than in controls: the fovea (272.10 ± 44.12 vs. 241.26 ± 34.75 μm; *p* = 0.001) and the nasal inner (NI) sector (333.62 ± 25.94 vs. 310.67 ± 18.13 μm; *p* = 0.001). The persistent foveal thickening likely reflects the known pattern of macular hole closure morphology, wherein centripetal approximation of retinal tissue results in residual foveal elevation and reorganization of inner retinal layers, including reparative gliosis [21, 22]. The increased NI thickness corroborates the nasal retinal displacement described after surgery with ILM peeling [12, 13]. 

GCC thickness (µm) was significantly reduced in all eight ETDRS sectors and in mean GCC thickness from the preoperative to the postoperative period (107.55 ± 11.80 to 95.97 ± 13.31 μm; *p* < 0.001). Postoperative mean GCC thickness remained significantly lower than in controls (95.97 ± 13.31 vs. 104.26 ± 7.95 μm; *p* = 0.001), suggesting persistent inner retinal layer compromise beyond what can be explained by the resolution of preoperative structural distortion alone.

The preoperative increase in GCC thickness relative to controls in most sectors likely reflects the architectural distortion, intraretinal cystoid changes, and redistribution of inner retinal layers secondary to vitreoretinal traction [23, 24]. The postoperative reduction in GCC thickness may therefore represent a combination of: (1) resolution of this preoperative structural distortion; (2) retinal remodeling following closure; and (3) potential neuronal or structural damage to the inner retinal layers resulting from the mechanical trauma of ILM peeling [7, 9, 25]. 

The significant increase in GCC thickness in the NI sector in postoperative patients compared to controls (127.05 ± 38.41 vs. 113.36 ± 8.12 μm; *p* = 0.033) is consistent with the pattern of nasal retinal displacement discussed above and parallels the NI sector finding for TMT [12, 13]. 

The sustained reduction in postoperative GCC thickness relative to normative values raises the possibility that ILM peeling may contribute to structural damage to the ganglion cell layer and inner plexiform layer that persists beyond the six-month follow-up period. Whether this structural change reflects true ganglion cell loss or is primarily a consequence of remodeling and redistribution of retinal components remains to be determined [23, 24, 26]. 

A central contribution of the present study was the topographic analysis of structure-function relationships between RS assessed by microperimetry and postoperative OCT thickness parameters across corresponding ETDRS sectors. Significant positive correlations between RS and TMT (µm) were found at the fovea (ρ = 0.542; *p* < 0.001), NI (ρ = 0.339; *p* = 0.033), and all four outer sectors (TO: ρ = 0.645; NO: ρ = 0.632; SO: ρ = 0.628; IO: ρ = 0.546; all *p* < 0.001), as well as for mean RS and mean TMT (ρ = 0.503; *p* = 0.001). The strongest correlations were consistently observed in the outer perifoveal sectors.

For GCC thickness (µm), significant correlations with RS were found in the TI (ρ = 0.391; *p* = 0.0003), TO (ρ = 0.369; *p* = 0.001), SO (ρ = 0.375; *p* = 0.001), and IO (ρ = 0.240; *p* = 0.029) sectors, as well as for mean values (ρ = 0.320; *p* = 0.003). These findings indicate that areas with greater structural preservation of both TMT and GCC exhibit better functional sensitivity, providing direct topographic evidence that inner retinal layer integrity is a meaningful determinant of local macular function after MH surgery.

Overall, structure-function correlations were more robust for TMT than for GCC thickness, likely reflecting the broader contribution of all retinal layers — including photoreceptors and the outer nuclear layer — to retinal light sensitivity. The observation that the strongest RS-TMT correlations occurred in the outer sectors, rather than at the fovea, is consistent with the functional role of the parafoveal and perifoveal retina in detecting spatial stimuli used in microperimetric testing [14, 15, 27]. 

The question of whether preoperative OCT structural parameters could serve as prognostic biomarkers for postoperative functional recovery is clinically important. The preoperative GCC and TMT values in this cohort (Tables 3 and 4) reflect primarily tractional distortion and intraretinal cystic changes rather than true structural preservation — making their interpretation as prognostic indicators complex. Prospective studies incorporating preoperative microperimetry alongside preoperative structural imaging and a dedicated prognostic modeling framework are warranted to address this question rigorously.

Preoperative BCVA showed significant inverse correlations with postoperative RS in the SI (*r* = − 0.394; *p* = 0.012), II (*r* = − 0.553; *p* = 0.0002) sectors, and mean RS (*r* = − 0.405; *p* = 0.010), indicating that worse preoperative visual function was associated with lower macular sensitivity at six months [30, 31]. 

The study cohort was predominantly composed of medium (50.0%) and large (22.5%) MH by MLD classification, with a mean MLD of 501.22 ± 176.65 μm. This distribution reflects the typical clinical profile encountered in surgical series and is consistent with the high closure rate achieved [4, 5]. 

Among the MH morphometric parameters assessed, a positive unadjusted correlation was observed between mean MLD and mean postoperative RS (*r* = 0.343; *p* = 0.040); however, this association did not survive Benjamini-Hochberg FDR correction. Stratified analysis by MH size category showed no significant difference in mean RS across size groups (Kruskal-Wallis, *p* > 0.05). This finding is most plausibly explained by a grid-averaging artifact inherent to the use of mean RS as a global metric: in eyes with smaller MH, deep central and juxtafoveal absolute scotomas substantially reduce mean sensitivity calculated across all 44 microperimetric points, while extrafoveal testing points — which constitute the majority of the grid — may be relatively preserved. This produces a spurious positive association between MH size and mean RS that is not replicated in sector-specific analyses. We therefore do not consider this a biologically meaningful prognostic signal [30]. 

In the multivariate analysis, temporal hole height was the only morphometric variable independently associated with postoperative foveal RS (β = −0.064; *p* = 0.024). When mean RS was used as the dependent variable, nasal hole height was the only predictor — among all morphometric and structural OCT parameters — to reach statistical significance (β = 0.054; *p* = 0.023); no sector-level GCC or TMT parameter was independently associated with mean RS after multivariate adjustment (*p* > 0.05 for all). This dissociation between the predictors of foveal RS and those of mean RS likely reflects the distinct determinants of central versus global macular sensitivity: foveal RS is influenced by the local structural environment of the perifoveal inner retinal layers, whereas mean RS integrates sensitivity across the full 44-point grid, diluting the contribution of any single structural sector. The nasal hole height finding is consistent with the pattern of nasal retinal displacement documented in this cohort, and highlights the potential prognostic relevance of vertical MH dimensions [31]. 

Multivariate regression analysis identified specific OCT sector thicknesses as independent predictors of postoperative foveal RS. For GCC thickness, significant associations were found in the SI (β = −1.471; *p* = 0.002), II (β = 0.550; *p* = 0.032), and SO (β = 0.550; *p* = 0.037) sectors. For TMT, independent associations were identified in the TI (β = −0.315; *p* = 0.001), II (β = 0.342; *p* = 0.001), and IO (β = −0.680; *p* = 0.001) sectors. Neither BCVA nor global mean GCC or TMT values reached significance in the multivariate model, underscoring that it is the regional distribution of structural changes — rather than global macular measures — that independently predicts local foveal function.

The coexistence of positive and negative β coefficients for adjacent ETDRS sectors warrants explicit clarification. Each β coefficient in a multivariate regression represents the *partial* conditional relationship between that predictor and the outcome after accounting for all other predictors simultaneously — not the global bivariate association. Adjacent ETDRS sectors are geometrically contiguous and therefore highly intercorrelated; when entered jointly into the model, shared variance is partitioned and residual conditional relationships can reverse sign relative to the bivariate correlations. This phenomenon — multicollinearity-induced sign reversal — is a recognized property of multivariate regression with correlated predictors. The bivariate Spearman correlations in Table 6 show uniformly positive associations between all OCT sectors and RS; the negative β coefficients in the multivariate models therefore do not imply biological antagonism but reflect statistical conditioning. Biologically, the mixed-sign pattern is also consistent with the spatial heterogeneity of postoperative macular remodeling: the NI sector shows paradoxical thickness increase relative to controls (reflecting nasal retinal displacement rather than structural preservation), and sectors undergoing tissue redistribution may bear a different structural-functional relationship to foveal sensitivity than sectors undergoing genuine atrophic thinning. For these reasons, the individual β coefficients should not be interpreted in isolation; the regression findings should be considered exploratory and hypothesis-generating.

These results extend and complement previous structure-function analyses by providing sector-by-sector topographic correspondence between OCT and microperimetric data — an approach applied in our group’s prior work on epiretinal membrane surgery [28, 29] and now extended to MH in the present series.

Several limitations of the present study should be acknowledged. First, the relatively modest sample size (*n* = 40) limits the statistical power of subgroup analyses and the generalizability of the multivariate models, which should be considered exploratory. Second, the cross-sectional design of the functional assessment, performed at a single postoperative time point (six months), precludes evaluation of longitudinal changes in RS and their relationship to structural remodeling over time. Third, the absence of a non-ILM peeling comparison group prevents direct attribution of the observed RS reduction to ILM peeling per se, as opposed to the natural course of MH-related damage. Fourth, while the matched control group enabled robust comparison of postoperative RS with normative values, preoperative microperimetric data were not available for patients, precluding direct pre-to-post functional comparison; prospective studies incorporating preoperative microperimetry are required to isolate the independent contribution of ILM peeling to functional outcomes. Fifth, the study was conducted at a single center and all procedures were performed by a single surgeon, which may limit external validity but ensures technical consistency. Sixth, the control group was required to have 20/20 BCVA, whereas postoperative MH eyes exhibited substantially worse BCVA (mean 0.54 ± 0.26 logMAR); although BCVA was included as a covariate in all regression models and did not emerge as a significant independent predictor of postoperative RS, the BCVA mismatch may contribute in part to the observed group differences in retinal sensitivity. Seventh, individual false-positive rates, false-negative rates, and fixation stability indices (P1, P2) were not systematically compiled and reported for each participant in this dataset; formal reporting of these metrics is recommended for future studies. Eighth, formal intra- and intergrader reproducibility analysis for OCT segmentation was not performed within this dataset. Ninth, the scatter plots in Fig. 5 pool sector-patient pairs for illustrative purposes and do not account for within-eye clustering of sectors; a mixed-effects model with patient-level random effects would be the appropriate approach for pooled sector-level analyses and is recommended for future studies.

The present findings carry important implications for the postoperative management of MH patients. The demonstration that significant RS deficits persist at six months despite successful anatomic closure and meaningful BCVA improvement argues for a broader definition of surgical success that encompasses functional parameters beyond visual acuity alone. Microperimetry, with its capacity for topographic, point-by-point structure-function correlation with OCT, represents a particularly valuable tool for this purpose [12, 14, 15]. 

Furthermore, the identification of GCC thinning as a correlate of reduced RS raises important questions about the long-term consequences of ILM peeling on inner retinal integrity. Strategies aimed at minimizing inner retinal trauma during ILM peeling — such as dye selection, peeling extent optimization, and instrument design — merit further investigation [7, 9]. 

## Conclusions

Despite successful anatomic MH closure and significant BCVA improvement, retinal sensitivity assessed by microperimetry remained significantly reduced across most extrafoveal ETDRS macular sectors at six months postoperatively. These functional deficits were accompanied by significant GCC thinning in sectors that were anatomically remote from the macular hole and structurally intact — indeed, exhibiting supranormal GCC thickness reflecting tractional distortion — prior to surgery. The spatial distribution of both functional and structural changes is incompatible with damage attributable to the macular hole itself, which in this series measured less than 1000 μm in all cases (mean MLD = 501 μm) and was confined to the foveal and immediately juxtafoveal zone. The postoperative reduction of GCC below normative values and the corresponding sensitivity deficits in anatomically distant extrafoveal sectors are therefore most plausibly explained by the mechanical trauma of ILM peeling. Significant topographic structure-function correlations between sector-specific RS and both TMT and GCC thickness across the ETDRS grid — with stronger associations in the outer perifoveal sectors — further support this interpretation. These findings support the integration of microperimetry as a complementary functional outcome measure in the postoperative assessment of macular hole surgery, and underscore the importance of strategies aimed at minimizing inner retinal trauma during membrane removal.

## Supplementary Information

Below is the link to the electronic supplementary material.

Supplementary Material 1: Figure 1. Macular hole (MH) size distribution and preoperative morphometric parameters assessed by optical coherence tomography (OCT). (A) Distribution of MH cases according to size classification based on minimum linear diameter (MLD). (B) Preoperative morphometric parameters including MLD, base diameter at the RPE level, nasal height, temporal height, and total hole height. Data are expressed as mean ± standard deviation. All measurements were obtained using the DRI OCT Triton (Topcon Corporation, Tokyo, Japan).

## Data Availability

No datasets were generated or analysed during the current study.

## Abbreviations

AUC: Area under the receiver operating characteristic curve
BCVA: Best-corrected visual acuity
BD: Base diameter
BCEA: Bivariate contour ellipse area
D: Diopter
dB: Decibel
DD: Disc diameter
DONFL: DONFL
ETDRS: Early treatment diabetic retinopathy study
FDR: False discovery rate
GCC: Ganglion cell complex
HOJF: Juiz de fora eye hospital
II: Inferior inner
ILM: Internal limiting membrane
IO: Inferior outer
IOP: Intraocular pressure
logMAR: Logarithm of the minimum angle of resolution
MH: Macular hole
MLD: Minimum linear diameter
NI: Nasal inner
NO: Nasal outer
OCT: Optical coherence tomography
PPV: Pars plana vitrectomy
PRL: Preferred retinal locus
RS: Retinal sensitivity
SI: Superior inner
SO: Superior outer
SPSS: Statistical package for the social sciences
TI: Temporal inner
TMT: Total macular thickness
TO: Temporal outer
UFJF: Federal university of juiz de fora
VA: Visual acuity
VIF: Variance inflation factor
µm: Micrometer
β: Standardized regression coefficient
ρ: Spearman correlation coefficient

## References

[1] Lois N, Burr JM, Norrie J, et al. Internal limiting membrane peeling versus no peeling for idiopathic full-thickness macular hole: a pragmatic randomised controlled trial. Invest Ophthalmol Vis Sci. 2011;52(3):1586–92. 10.1167/iovs.10-6287 21051731

[2] Christensen UC. Value of internal limiting membrane peeling in surgery for idiopathic macular hole and the correlation between function and retinal morphology. Acta Ophthalmol. 2009;87(Thesis 2):1–23. 10.1111/j.1755-3768.2009.01777.x 19912135

[3] Kwok AK, Lai TY, Wong VW. Idiopathic macular hole surgery in Chinese patients: a randomised study to compare indocyanine green-assisted internal limiting membrane peeling with no internal limiting membrane peeling. Hong Kong Med J. 2005;11(4):259–66. 16085942

[4] Manvikar SR, Steel DH. Internal limiting membrane removal during macular hole surgery. Ophthalmology. 2006;113(8):1303–9. 10.1016/j.ophtha.2006.02.061 16877079

[5] Schneider EW, Mruthyunjaya P. Internal limiting membrane peel size and macular hole surgery outcome. Eye (Lond). 2025. 10.1038/s41433-025-03666-9. PMC12044072 39922971

[6] Gelman R, Stevenson W, Prospero Ponce C, Agarwal D, Christoforidis JB. Retinal damage induced by internal limiting membrane removal. J Ophthalmol. 2015;2015:939748. 10.1155/2015/939748 PMC4573889 26425355

[7] Tadayoni R, Paques M, Massin P, et al. Dissociated optic nerve fiber layer appearance of the fundus after idiopathic epiretinal membrane removal. Ophthalmology. 2001;108(12):2279–83. 10.1016/s0161-6420(01)00856-9 11733271

[8] Ito Y, Terasaki H, Takahashi A, et al. Dissociated optic nerve fiber layer appearance after internal limiting membrane peeling for idiopathic macular holes. Ophthalmology. 2005;112(8):1415–20. 10.1016/j.ophtha.2005.02.023 16061095

[9] Haritoglou C, Gass CA, Schaumberger M, et al. Macular changes after peeling of the internal limiting membrane in macular hole surgery. Am J Ophthalmol. 2001;132(3):363–8. 10.1016/s0002-9394(01)01093-5 11530049

[10] Haritoglou C, Ehrt O, Gass CA, Kristin N, Kampik A. Paracentral scotomata: a new finding after vitrectomy for idiopathic macular hole. Br J Ophthalmol. 2001;85(2):231–3. 10.1136/bjo.85.2.231 PMC1723828 11159494

[11] Tadayoni R, Svorenova I, Erginay A, Gaudric A, Massin P. Decreased retinal sensitivity after internal limiting membrane peeling for macular hole surgery. Br J Ophthalmol. 2012;96(12):1513–6. 10.1136/bjophthalmol-2012-302035 PMC3512349 23077227

[12] Imai H, Iijima H, Morizane Y, et al. Changes in retinal sensitivity and fixation stability after idiopathic macular hole surgery evaluated with microperimetry. Retina. 2018;38(10):2000–7.

[13] Baba T, Yamamoto S, Arai M, et al. Temporal thinning of the retina after internal limiting membrane peeling for macular hole. Ophthalmology. 2014;121(1):215–22.

[14] Midena E, Vujosevic S. Microperimetry in retinal diseases. Middle East Afr J Ophthalmol. 2023;30(1):2–11.

[15] Springer C, Bültmann S, Völcker HE, Rohrschneider K. Functional vs morphologic criteria of macular holes: a microperimetric and optical coherence tomography study. Am J Ophthalmol. 2005;140(4):588–94.

[16] Lai TT, Yang CM, Yang CH. Changes in retinal sensitivity after successful macular hole surgery evaluated by microperimetry. Retina. 2019;39(5):949–56.

[17] Sborgia G, Niro A, Sborgia L, et al. Microperimetric changes after internal limiting membrane peeling for macular hole surgery. Int Ophthalmol. 2020;40:2427–35.

[18] Kumagai K, Ogino N, Furukawa M, et al. Retinal thickness after vitrectomy and internal limiting membrane peeling for macular hole and epiretinal membrane. Clin Ophthalmol. 2012;6:679–88. 10.2147/OPTH.S30288 PMC3363315 22654493

[19] Ooto S, Hangai M, Takayama K, et al. Macular structure after successful macular hole surgery analyzed by spectral-domain OCT. Ophthalmology. 2011;118(7):1260–7.

[20] Liu L, Wang Y, Li Y, et al. Retinal layer thickness changes after successful idiopathic macular hole surgery measured by spectral-domain OCT. Graefes Arch Clin Exp Ophthalmol. 2016;254:1417–24.

[21] Spaide RF. Dissociated optic nerve fiber layer appearance after internal limiting membrane removal is inner retinal dimpling. Retina. 2012;32(9):1719–26. 10.1097/IAE.0b013e3182671191 23007669

[22] Ishida M, Shiode Y, Morizane Y, et al. Inner retinal layer thickness changes after internal limiting membrane peeling for idiopathic macular hole. Am J Ophthalmol. 2018;187:130–9.

[23] Lee H, Chung H, Kim HC. Changes in ganglion cell-inner plexiform layer thickness after successful macular hole surgery. Invest Ophthalmol Vis Sci. 2018;59(8):3234–40.

[24] Bacherini D, Caporossi T, Dragotto F, et al. Ganglion cell complex analysis after macular hole surgery using spectral-domain OCT. Int Ophthalmol. 2021;41:3659–67.

[25] Pichi F, Lembo A, Morara M, et al. Early and late inner retinal changes after internal limiting membrane peeling. Int Ophthalmol. 2014;34(2):437–46. 10.1007/s10792-013-9831-6 23864285

[26] Iwasaki M, Kinoshita T, Miyamoto H, et al. Long-term outcomes after vitrectomy with internal limiting membrane peeling for idiopathic macular hole. Am J Ophthalmol. 2017;179:38–44.

[27] Inoue M, Watanabe Y, Arakawa A, Kadonosono K. Structure-function correlation using optical coherence tomography and microperimetry after idiopathic macular hole surgery. Sci Rep. 2022;12:18952.

[28] Matos AMF, Defina RLS, Costa-Cunha LVF, et al. Correlation between retinal sensitivity assessed by microperimetry and structural abnormalities on optical coherence tomography after successful epiretinal membrane surgery. Int J Retina Vitreous. 2024;10(1):21. 10.1186/s40942-024-00542-8. PMC10905782 38424619

[29] Cunha LP, Matos AMF, Defina RLS, et al. Assessment of ganglion cell complex thickness and its correlation with retinal sensitivity using microperimetry 6 months after epiretinal membrane surgery. Int J Retina Vitreous. 2024;10(1):58. 10.1186/s40942-024-00576-y. PMC11344420 39180080

[30] Mehta N, Simonett J, Lee BJ. Assessing the ability of preoperative spectral-domain OCT characteristics to predict visual outcome in idiopathic macular hole surgery. Retina. 2021;41(4):742–9. 10.1097/IAE.0000000000002797 PMC7529763 32251240

[31] Lorenc O, Safranow K, Machalińska A. Predictive value of preoperative anatomical and functional parameters for long-term visual outcomes after full-thickness macular hole surgery. J Clin Med. 2025;14:1263. 10.3390/jcm14248757 PMC12733625 41464659

